# Research into Autonomous Vehicles Following and Obstacle Avoidance Based on Deep Reinforcement Learning Method under Map Constraints

**DOI:** 10.3390/s23020844

**Published:** 2023-01-11

**Authors:** Zheng Li, Shihua Yuan, Xufeng Yin, Xueyuan Li, Shouxing Tang

**Affiliations:** National Key Laboratory of Vehicular Transmission, Beijing Institute of Technology, Beijing 100081, China

**Keywords:** autonomous driving, deep reinforcement learning, car following, obstacle avoidance, obstacle representation

## Abstract

Compared with traditional rule-based algorithms, deep reinforcement learning methods in autonomous driving are able to reduce the response time of vehicles to the driving environment and fully exploit the advantages of autopilot. Nowadays, autonomous vehicles mainly drive on urban roads and are constrained by some map elements such as lane boundaries, lane driving rules, and lane center lines. In this paper, a deep reinforcement learning approach seriously considering map elements is proposed to deal with the autonomous driving issues of vehicles following and obstacle avoidance. When the deep reinforcement learning method is modeled, an obstacle representation method is proposed to represent the external obstacle information required by the ego vehicle input, aiming to address the problem that the number and state of external obstacles are not fixed.

## 1. Introduction and Backgrounds

In a mature autonomous driving system, the environment perception module is equivalent to human eyes and ears, while the decision-planning module is equivalent to the human brain.

Most traditional decision-planning modules have a layered and hierarchical architecture, where the content is transferred layer by layer. Such a structure makes it easier to decompose complex tasks; thus, it is convenient to detect problems with the specific module. In addition, relying heavily on prior knowledge and formulae, traditional methods have strong interpretability. Owing to their good performance in terms of accuracy and interpretability, the traditional decision-planning modules are the mainstream system of autonomous driving technology at present. It is indeed established that the cascade structure in traditional approaches also brings drawbacks in the aspects of instantaneity and reliability. First, data computation and transmission through different layers hinder the instantaneity and flexibility of the modules. Second, the operation of traditional modules requires all elements in it to operate under normal condition, or it will be out of order, i.e., the system of traditional decision-planning modules is vulnerable and unreliable.

The adaptive cruise control (ACC) method has been widely applied in most traditional vehicle-following practices. In order to maintain a relatively stable distance with the target vehicle, the speed of the ego vehicle changes along with the target one on the straight road. As described in [1], the paper used the SMPC method, and a conditional linear Gaussian model was proposed and trained with actual measurements to estimate the probability distribution of the future speed of the preceding vehicle. Then, the paper built the relevant driving scenarios in the single lane in the CarMaker, applied the control strategy in the simulation scene, and verified it. Ioannou et al. modeled the ACC system as a hybrid system with constant acceleration, smooth acceleration, and a linear state feedback controller in [2]. Kitazono et al. in [3] adopted an adaptive neural network scheme to solve the traffic stability problem in convoys. In addition, there are also many other different strategies used to adjust the distance between the ego and target vehicles, with the utilization of the ACC system. In [4], Choi et al. used a fixed distance strategy and a sliding mode control method to control the speed and distance between two vehicles.

There are two main traditional obstacle avoidance methods, including reference line fitting methods and artificial potential field methods. As for the reference line fitting method, the rationale of trajectory planning is widely adopted to generate a cluster of alternative trajectories, with the consideration of vehicle dynamics and the general vehicle attitudes. Then, these trajectories are sorted on the basis of cost value, and a collision-free obstacle avoidance trajectory with the least cost is finally obtained after collision detection. Finally, the trajectory tracking control algorithm is used to control the ego vehicle to avoid the obstacles. The artificial potential field method is a virtual force method, and its basic idea is to regard the vehicle’s motion in the surrounding environment as the motion of the vehicle in the artificially established virtual position. The target point generates gravity, which guides the vehicle toward the target point, and the obstacles generate repulsion to avoid a collision between the ego vehicle and the obstacles. Under the combined force of gravity and repulsion, the ego vehicle moves along the direction of the potential field decline, resulting in an optimal path without collision. To avoid the local minimum, Ref. [5] proposed a virtual potential field detection circle model with an adjustable radius. The “minimum trap” formed by the obstacle repulsion field had been detected by the model in advance, whose success rate in obstacle avoidance has reached 90% or more. In [6], the obstacle avoidance function was introduced into the path planning layer, and the trajectory tracking layer transformed the linear time-varying MPC optimization problem into a quadratic programming problem, which has realized the path emergency obstacle avoidance and tracking.

Recently, some optimization methods based on traditional obstacle avoidance have been proposed and have achieved good results. In [7], a personalized motion planning and tracking control framework was proposed to prevent collisions between autonomous vehicles and obstacles ahead. The simulation results showed that the proposed framework could improve the corresponding driving performance according to the individualized needs of different passengers. The author in [8] improved an algorithm integrating path pruning, smoothing, and optimization with geometric collision detection based on the rapidly exploring random trees (RRT) algorithm. The simulation indicated that the vehicle could successfully track the path efficiently and reach the destination safely. An improved heuristic Bi-RRT algorithm, specialized for an unknown dynamic environment, was put forward by [9] for obstacle avoidance. Thence, the related experiments have verified the good performance of such an algorithm in differentiable coherence path generation, ensuring both ride comfort and stability of the vehicle.

Today, with the continuous development of artificial intelligence technology, more industries have entered the digital deepening and intelligent extension stage. The essence of digitization is data, while the foundation of intelligence is artificial intelligence technology. In the current stage of autonomous driving, technology based on data-driven methods has been fully developed. Until now, data-driven methods have not only played a part in the perception and positioning of autonomous driving technology, but have also imposed increasing influences in decision-making planning and autonomous driving control technology.

Reinforcement learning is a machine learning method, often used to deal with sequential decision-making issues. Since the reinforcement learning method is trained through trial and error in the training process, the method strongly depends on the simulation environment. For the autonomous driving decision-planning module based on the reinforcement learning method, the system’s input can be raw perceptual data, such as pixel data from an RGBD camera or lidar points cloud data. Refs. [10,11,12,13] presented some model-free deep reinforcement learning methods for autonomous driving. For those data with high dimensions, a convolutional neural network for dimensionality reduction is necessary before being passed into the reinforcement learning network. Being mature, the external perception and recognition technology based on cameras and lidars make accurate data accessible, including the ego surrounding environment status information, target vehicle status information, and self-vehicle status information. Moreover, developers can directly obtain a list of relevant objects from the simulation platform.

Recently, reinforcement learning algorithms have been increasingly utilized to deal with complex sequential decision-making and control problems. In the following field of vehicles, the literature [14] applied the policy gradient algorithm in reinforcement learning to train the CACC longitudinal controller to eliminate the errors of car following. However, the exploitation of the discrete control variable and more straight-forward environmental reward rules caused the repeated oscillations of the control quantities during the simulation. Ref. [15] proposed a potential game-combined multi-agent deep deterministic policy gradient (MADDPG) approach to optimize multiple UAVs’ trajectories. Ref. [16] put forward a method that combined imitation learning with reinforcement learning enabling the agent to achieve a higher success rate in urban autonomous driving navigation scenarios. The literature [17] designed an autonomous driving system using the Q network and A* algorithm. For an unknown rough terrain scenario, Ref. [18] constructed a deep reinforcement learning method for safe local planning of a ground vehicle. However, the above-mentioned algorithms were still restricted within the longitudinal control on straight roads, but seldom addressed the issues related to the turning part of intersections.

Similarly, in the obstacle avoidance part of the high-precision map, obstacle avoidance methods are all based on reinforcement learning methods. The literature [19] investigated the dual-target behavior of trajectory tracking and obstacle avoidance based on waypoints through augmenting the agent’s observation vector by the reward trade-off parameter, thus enabling the agent to adapt to changes in its reward function. In [20], the author proposed a deep Q-network-based method for collision-free decisions. The DQN-aided algorithm was introduced for determining the trajectory and velocity of the AV by receiving real-time traffic information from the base stations. In [21], they presented a novel learning-based control algorithm for ASV systems with collision avoidance. The proposed control algorithm combined a conventional control method with deep reinforcement learning to provide a closed-loop stability guarantee, uncertainty compensation, and collision avoidance. The scholars in [22] proposed an interpretable decision-making framework for autonomous vehicles at highway on-ramps adopting a latent space actor–critic-based method with asymmetric inputs. When constructing the simulation scene, the ego vehicle and the obstacle are usually simplified as a circle or a mass point, where the circle center is the center of the vehicle, and the radius is the safety distance. In this case, the interaction between the vehicle’s polys (i.e., the attitude requirements of the vehicle) has been ignored, especially when passing through a narrow passable area. Hence, such methods did not make full use of the advantages of reinforcement learning. Meanwhile, the reinforcement learning method requires the input dimension of the object list to be unified. Such requirements have imposed more challenges to the scene where the external obstacles are not fixed.

Based on the above-mentioned problems ignored in previous studies on autonomous driving related to reinforcement learning, this paper conducts related research using the CARLA simulation platform. CARLA is an open-source autonomous driving simulation platform based on the UE4 engine’s rendering, which provides users with the Python API interface, which can build rich driving scenes, and rich map elements can be obtained. In addition, the CARLA platform can also import self-built OpenDRIVE map files and OpenSCENARIO autonomous driving scene files, which can be customized according to the users’ own needs.

In this paper, the factor of the braker is not taken into consideration in the CARLA simulation platform, while only two control quantities are required in the controlled vehicle, i.e., the steering wheel angle and the accelerator pedal strength. Previous studies on autonomous driving technology based on reinforcement learning usually discretize both control quantities, with the utilization of the reinforcement learning model based on the DQN method for training. From a human driver’s perspective, when driving a vehicle, the steering wheel angle and the brake pedal’s position can take any value within the limit range. In other words, the input variables are continuous. To give full play to the characteristics of the neural network model and get closer to the level of human driver operation, the reinforcement learning basic model in this paper adopts the twin delayed deep deterministic policy gradient network framework that can generate actions in a continuous space. Additionally, this method will be referred to as TD3 for short. To sum up, the relevant scenarios of car following and obstacle avoidance in this paper are all carried out using the CARLA simulation platform, and the TD3 method is used as the calculation benchmark. The main contributions of this article are as follows:

- A car-following model method based on reinforcement learning is proposed, which combines the relevant map elements in the map and the Frenet coordinate system, and can follow the car stably on straights, intersections, and curves.
- We propose an external obstacle state input algorithm that can be adaptive to the ego vehicle’s speed. Additionally, this algorithm can represent the shape and pose of a random number of obstacles.
- An obstacle avoidance method using reinforcement learning under map constraints is designed based on the improved obstacle input method. This method can still perform well when passing through narrow passable areas.

The rest of this paper is organized as follows. In Section 2, a reinforcement learning-based target tracking model is presented, and this model can have perfect curve passing performance. Section 3 mainly introduces a multi-obstacle representation method. Finally, Section 4 concludes the paper.

## 2. Reinforcement-Learning-Based Car following Method Using the Map Constraints

### 2.1. Reinforcement Learning Theory

To meet the continuous demand of action space for policy optimization, this paper selects the TD3 as the basic algorithm model based on the actor–critic structure.

The actor–critic algorithm is an extension of the policy gradient method. In its single-step updated calculation, the core formula can be expressed as follows:

(1) $$\{\begin{array}{c} \delta =r_{t}\left(s_{t},a_{t}\right)+v_{\omega }\left(s_{t+1}\right)-v_{\omega }\left(s_{t}\right)\\ \omega_{t+1}=\omega_{t}+\alpha^{\omega }\delta_{t}{𝛻}_{\omega }v_{\omega }\left(s_{t}\right)\\ \theta_{t+1}=\theta_{t}+\alpha^{\theta }{𝛻}_{\theta }\log\pi_{\theta }\left(a_{t}|s_{t}\right)\delta_{t} \end{array}$$

Among them, the first line is the calculated TD error, the second line is the updated critic model parameters, and the third line is the updated actor model parameters.

#### TD3 Theoretical Knowledge

Google DeepMind combines the above actor–critic method with the DQN network and proposes a deep deterministic policy network (deep deterministic policy gradient), referred to as DDPG. The calculation formula of its objective function is as follows:

(2) $$\{\begin{array}{c} J\left(\omega \right)=min_{\omega }E_{\beta }\left[\frac{1}{2}{\left(r_{t}+\gamma Q^{\omega }\left(s_{t+1},a_{t+1}\right)-Q^{\omega }\left(s_{t},a_{t}\right)\right)}^{2}\right]\\ J\left(\theta \right)=max_{\theta }E_{\beta }\left[Q^{\omega }\left(s_{t},\mu \left(s_{t}\right)\right)\right] \end{array}$$

Among them, ω is the critic network parameter, θ refers to the actor network parameter, Q(s,a)  is the action value function, μ(s)  is the deterministic strategy, and γ is the discount factor. By solving the above objective function, we can obtain

(3) $$\{\begin{array}{c} \Delta \omega =E_{\beta }\left[\left(r_{t}+\gamma Q^{\omega }\left(s_{t+1},a_{t+1}\right)-Q^{w}\left(s_{t},a_{t}\right)\right){𝛻}_{\omega }Q^{\omega }\left(s_{t},a_{t}\right)\right]\\ \Delta \theta =\alpha E_{\beta }\left[{𝛻}_{\omega }Q^{\omega }\left(s_{t},a_{t}\right){|}_{a=\mu_{\theta }\left(s\right)}𝛻\mu_{\theta }\left(s_{t}\right)\right] \end{array}$$

The TD3 method in this paper is called the twin delayed deep deterministic policy gradient, which has been improved as follows based on DDPG:

(1) Actor network and critic network delayed asynchronous updates. After updating the critic network several times, update the actor network to ensure that the training of the actor network is more stable.

The actor network will be updated to ensure its stability with regard to the training performance.

(2) Two sets of twin critical networks are used, and the smaller value is selected in the actual calculation to prevent the network from overestimating.

(3) Adding perturbations to the actor’s internal network output $\;\varepsilon_{2}\;$ makes the target network estimate more accurately and robustly.

**Figure 1:**
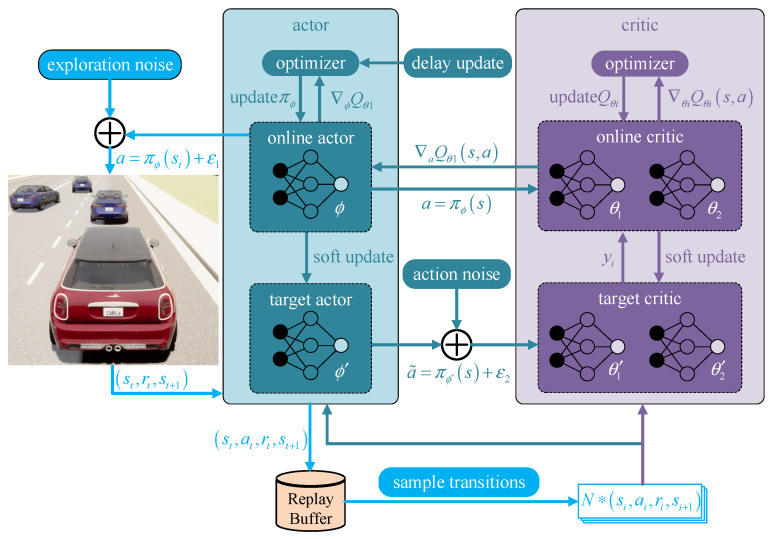
The improved TD3 network in this paper.

The structure of the TD3 network is constructed in Figure 1:

Combined with the reinforcement learning TD3 structure and the simulation process performance used in training, data units are stored in the container named replay buffer during the entire training process. This paper adopts a method that dynamically adjusts the replay buffer’s size according to different training stages, which is divided into three stages [23]. The first stage refers to the process of data storage, whose capacity is small. Therefore, it is easy to reach the upper limit and enter the second stage, which refers to the process of storing data while training. In this stage, the capacity of the replay buffer is also increased until the third stage is entered. In the third stage, the final replay buffer capacity is reached, but the data volume does not increase at this stage. Whenever new data are added, the farthest data will be popped out from the replay buffer to ensure the constancy of the replay buffer’s capacity.

### 2.2. State Function and Reward Function

#### 2.2.1. Frenet Coordinate System Introduced

**Figure 2:**
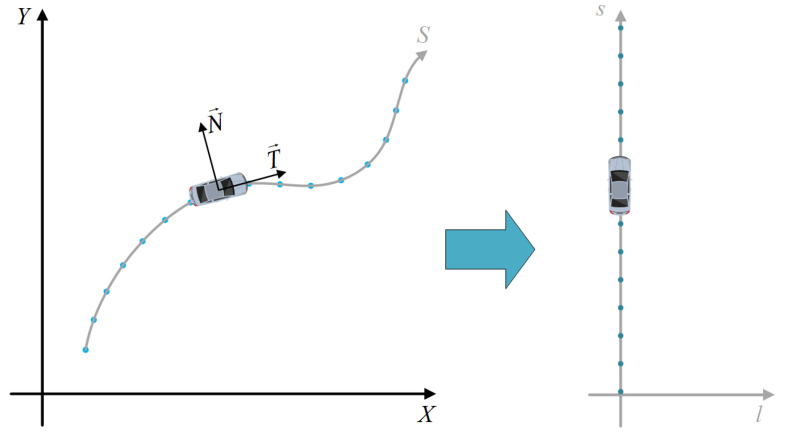
Comparison of the trajectory along the reference line in the Cartesian coordinate system and the Frenet coordinate system.

In urban structured roads, one of the challenges in the path planning module of autonomous driving technology is to continuously express the relative position between the vehicle and the road map during the whole driving process, resulting in a fuzzy relative position relationship between them. If the lane information is ignored, the degree of freedom of the path will be too high to control so that it is easy to violate road traffic rules. During the DARPA Automotive Challenge, Stanford University proposed the Frenet coordinate system, as shown in Figure 2, in which s represents the distance along the road and l represents the displacement from the longitudinal line.

**Figure 3:**
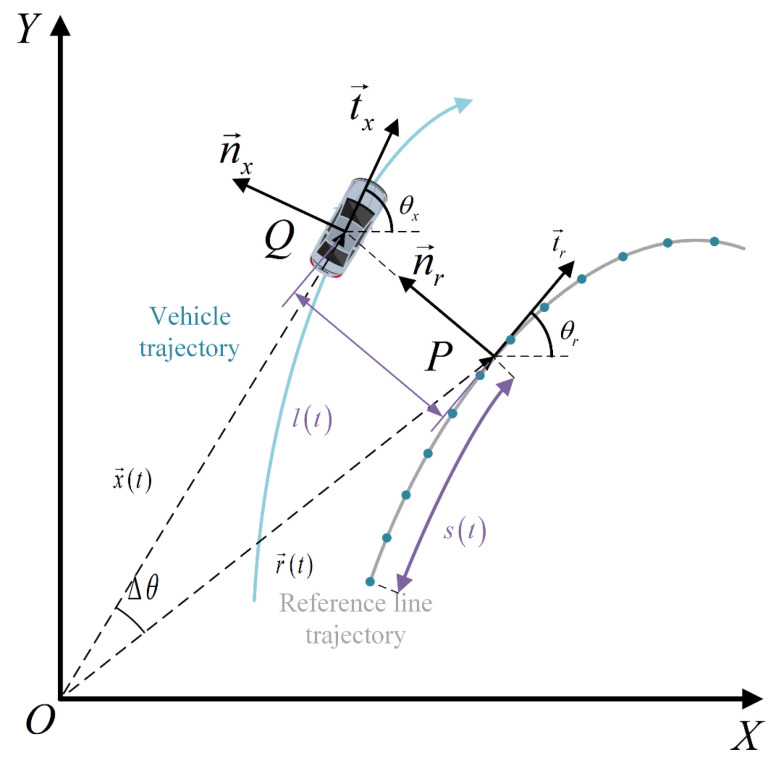
Schematic diagram of Frenet coordinate system and Cartesian coordinate system in normal driving state.

If the vehicle drives along the lane (usually the lane center reference line), in the Cartesian coordinate system the trajectory of the vehicle is consistent with the reference line, as shown on the left of the above figure. In the Frenet coordinate system, if the vehicle drives along the lane, the trajectory becomes a straight line traveling along s, as shown on the right of the above figure, which greatly simplifies the difficulty of trajectory calculation. In normal driving scenarios, the trajectory of the vehicle and the reference line usually do not overlap. As shown in Figure 3, the blue one is the actual driving trajectory of the vehicle, while the dark gray one represents the reference line trajectory, and the points on the reference line are waypoints. In the global coordinate system, the motion state of the ego vehicle at any time t can be described as $\left[\vec{x},\theta_{x},v_{x},a_{x}\right]$, in which $\vec{x}$ represents the current position of the vehicle marked as Q, and in the global Cartesian coordinate system it is represented as $\vec{x}\left(t\right)=\left(x\left(t\right),y\left(t\right)\right)$. In the Frenet coordinate system, P is the projection point of the current position point Q on the reference line, s refers to the arc length of the road lane from the starting point of the reference line to the projection point P, and l is the distance between the Q point and the projection point P, so it can be described by the longitudinal displacement s and the lateral displacement l relative to the reference line, i.e., $\vec{x}\left(t\right)=\left(x\left(t\right),y\left(t\right)\right)=\left(s\left(t\right),l\left(t\right)\right)$. In the actual code, the position of the projected point P is calculated by the two closest waypoints to the *Q* point.

#### 2.2.2. Environmental Scenario Analysis

**Figure 4:**
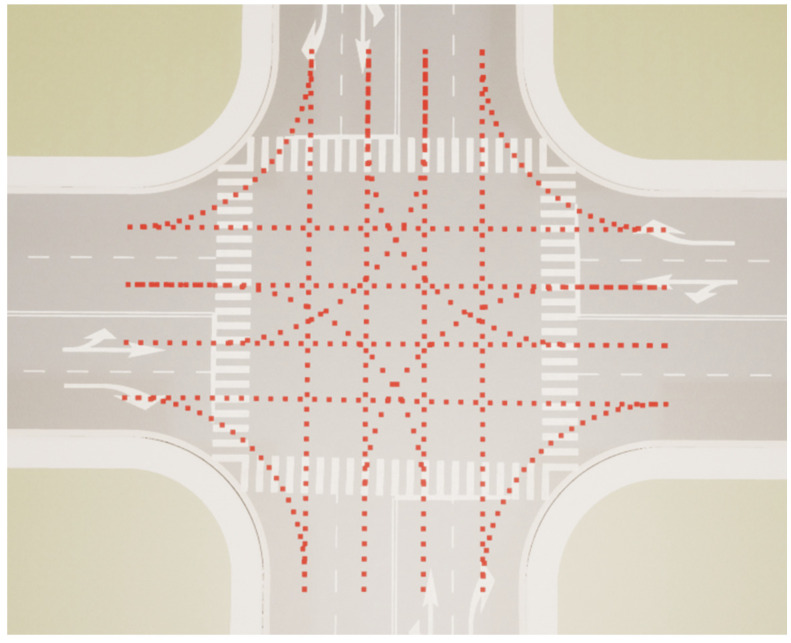
Waypoints at the road junction.

When the ego vehicle follows the target vehicle ahead at a non-junction position such as a straight road or a one-way road, the reference waypoint is on the center line of each lane, and the tracking exercise will be simpler. At the road junction, the distribution of waypoints is relatively dense, and the calculated closest point may be different from the lane where the preceding vehicle is located, as shown in the following Figure 4.

Therefore, when building the environmental scene and driving scenario in this paper, the driving options (straight, turn left, turn right) of the preceding vehicle at the junction should be confirmed first according to the change in the heading angle of the preceding target vehicle, and then a junction option list can be obtained. The ego vehicle reference line is updated according to the target junction option set. The detailed algorithm steps are presented as Algorithm 1.
**Algorithm 1.** Reference waypoints’ set acquisition algorithm in each episodeInitialize the ego vehicle reference waypoint list.Initialize the target vehicle steering option list.Use the nearest waypoint from the CARLA map to the location of the ego car as the current waypoint.**Repeat** (for each episode): **while** the size of waypoints is less than N **do**:  Add the waypoint list to the current waypoint, and find the next waypoint at the interval of sampling_dis according to the current waypoint.  If there is more than one choice for the next waypoint area, the next waypoint will be chosen randomly.  Set the new next waypoint as the new current waypoint.  Update the steering option of the target vehicle at the intersection to the waypoint list.  If the waypoint of the ego vehicle reference waypoint list is the first waypoint at the junction, update the waypoint according to the target steering option list.  Re-update the ego vehicle reference waypoint list.  When the ego vehicle exits the corresponding junction, pop the target vehicle steering option list. **end while**

#### 2.2.3. State Function

The set list of ego vehicle reference waypoints is made of all waypoints on the center line of the lane, which are equivalent to a reference line. According to the content of the previous section, the position of the ego vehicle can be projected onto the correct lane by using the set list of reference waypoints so as to obtain the longitudinal distance s and lateral distance l of the ego vehicle relative to the reference line.

Therefore, under the scene without map constraints, the ego vehicle’s and target’s relative state information can be utilized. However, when there are map constraints, and the state input should be considered, this paper selects the relative state of the ego vehicle and the target vehicle as $S_{t}^{ego\_obj}$, as well as the relative state of the ego vehicle and the reference waypoint set list as $S_{t}^{ego\_wps}$.

(4) $$S_{t}=\left\{S_{t}^{ego\_obj},S_{t}^{ego\_wps}\right\}$$

When considering the relative relationship between the ego vehicle and the target vehicle, different from the reinforcement learning method without map constraints, it needs to be calculated in the Frenet coordinate system as follows:

(5) $$\begin{array}{c} S_{t}^{ego\_obj\_1}=long\_dis_{obj}-long\_dis_{ego}\\ S_{t}^{ego\_obj\_2}=clip\left(\frac{vel_{ego\_long}}{clip\left(vel_{obj\_long},min,max\right)},min,max\right)\\ S_{t}^{ego\_obj}=S_{t}^{ego\_obj\_1}+S_{t}^{ego\_obj\_2} \end{array}$$

Among them, $S_{t}^{ego\_obj\_1}$ represents the distance between the ego vehicle and the target vehicle, i.e., the integral arc length between the projection points of the ego vehicle and the preceding vehicle on the lane. Under the constraints of the map lane, this state can more accurately reflect the relative position of the ego car and the preceding target vehicle than the line segment distance between the ego and target vehicles. $S_{t}^{ego\_obj\_2}$ is the ratio of the projection of the speed of the ego car and the target car in the Frenet coordinate system in the *s* direction. The closer the ratio $S_{t}^{ego\_obj\_2}$ is to 1, the closer the speed components of the ego car and the target vehicle are. The function of clip() in the formula is to intercept the value, which can prevent the denominator from being zero or the ratio from being too large and blocking within the boundary range.

When considering the relative relationship between the ego vehicle and the set of waypoints, the lateral distance between the ego vehicle, and the lane center line, the yaw angle deviation between the ego vehicle and the corresponding waypoint are mainly analyzed, as shown in the following formula

(6) $$\begin{array}{c} S_{t}^{ego\_wps\_1}=lateral\_dis_{obj\_wps}\\ S_{t}^{ego\_wps\_2}=yaw_{ego}-yaw_{wp} \end{array}$$

In addition, the state function also introduces preview points, i.e., three waypoints in the direction of the vehicle’s forward direction in the lane center line. The difference between these three preview waypoints and the physical quantities related to the vehicle is expressed as follows:

(7) $$S_{t}^{ego\_wps\_3}=\left[\begin{array}{ccc} wp\_1_{x}-ego_{x} & wp\_1_{y}-ego_{y} & wp\_1_{yaw}-ego_{yaw}\\ wp\_2_{x}-ego_{x} & wp\_2_{y}-ego_{y} & wp\_1_{yaw}-ego_{yaw}\\ wp\_3_{x}-ego_{x} & wp\_3_{y}-ego_{y} & wp\_1_{yaw}-ego_{yaw} \end{array}\right]$$

Therefore, the relative state of the ego vehicle and the set of reference waypoints $S_{t}^{ego\_wps}$ can be expressed as follows:

(8) $$S_{t}^{ego\_wps}=S_{t}^{ego\_wps\_1}+S_{t}^{ego\_wps\_2}+S_{t}^{ego\_wps\_3}$$

In the external interface of the CARLA platform, three variable parameters control the driving of the vehicle: the straight-forward parameter, the steering parameter, and the braking parameter. For the scene in this section, the braking parameters are not considered for the time being. Therefore, the outputs of the entire neural network contain the straight direction control command and the steering direction control command, which are consistent with the model without map constraints. To make the action value acting in the simulation environment smoother, the two variables directly outputted from the neural network are adjusted as follows and then directly act on the CARLA simulation platform:

(9) $$\begin{array}{c} a_{throttle}^{t+1}=a_{throttle}^{t}+\gamma_{1}\left(a_{throttle}^{t+1}-a_{throttle}^{t}\right)\\ a_{steer}^{t+1}=a_{steer}^{t}+\gamma_{2}\left(a_{steer}^{t+1}-a_{steer}^{t}\right) \end{array}$$

$\gamma_{1}$ and $\gamma_{2}$ are weight values from 0 to 1. It can be seen that the smoothed current time variable is the weighted value of the variable directly outputted by the neural network at the last time and the current time.

In the episode-based training process of reinforcement learning, if some policy solutions with relatively poor effects cannot be terminated in time, the invalid training may waste resources and time. Therefore, the reinforcement learning episode exit condition in this paper involves multiple termination trigger conditions.

**Figure 5:**
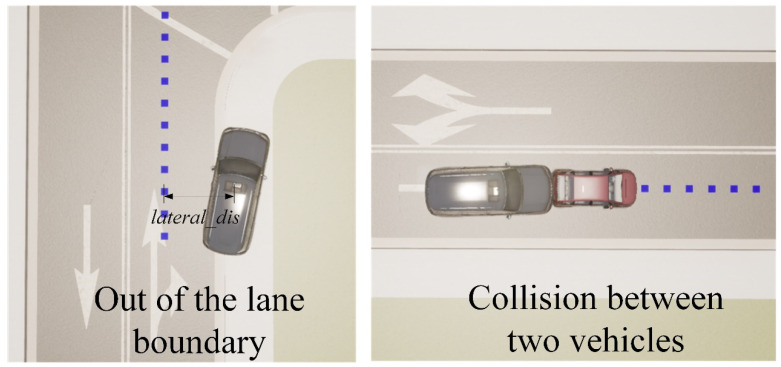
Two scenarios for exiting training episodes.

(1) Among the trigger conditions, the most important and intuitive termination condition is the occurrence of a collision event. Safety is undoubtedly the most important thing for self-driving vehicles. A collision detector can be added in CARLA. When the ego vehicle collides with other objects, a collision signal is generated. Within a period of the round, once the collision signal is detected, the current round will exit immediately. The relevant scenario is shown on the left side of Figure 5.

(2) The lane departure event is also a relatively intuitive termination condition. In the early stage of training, the neural network training is not stable, and the trajectory of the vehicle sometimes deviates too far from the correct lane; thus, a restriction needs to be added. Combined with the physical quantity of the lateral offset of the vehicle lane center line point set in the above state factors, the maximum value of the lateral offset is specified. When the lateral offset exceeds the threshold, the termination condition will be triggered, and the current round will exit immediately. This scenario is shown on the right side of Figure 5.

(3) The neural network outputs two control commands, namely the accelerator command and the steering wheel command. In the early stage of training, the speed of the ego vehicle may be too low or even close to zero. Therefore, a minimum speed threshold is specified. When the speed of the ego vehicle is lower than the threshold for several consecutive steps, the termination condition will be triggered and the current episode will exit.

(4) Ideally, during an episode of training, the ego vehicle will not meet the above-mentioned exit conditions, but it cannot be stuck in a certain episode forever and cannot exit. Therefore, in this paper, the maximum number of training steps is added in the episode training process. When the number of training steps in the episode reaches the maximum number of steps, the termination condition will be triggered and the current episode will exit.

#### 2.2.4. The Design of the Reward Function

As mentioned above, in addition to the influence of the relative position between two vehicles with regard to the reward, when designing the reward function, the influence of the set of lane center line points in the map and the relative attitude of the ego vehicle with regard to the reward function should also be considered. The design of the reward function corresponds to the above state function. For the set of waypoints, the reward function is as follows:

(10) $$\begin{array}{c} r_{t}^{ego\_wps\_1}=k_{1}\left|lateral\_dis_{obj\_wps}\right|+b_{1}\\ r_{t}^{ego\_wps\_2}=k_{2}\left|yaw_{ego}-yaw_{wp}\right|+b_{2}\\ r_{t}^{ego\_wps}=r_{t}^{ego\_wps\_1}+r_{t}^{ego\_wps\_2} \end{array}$$

Among them, $k_{1}$, $k_{2},$ and $b_{1}$, $b_{2}$ are the artificially designed algorithm parameters. In actual training, the farther the ego vehicle deviates from the center of the lane, the smaller the reward value; the larger the yaw difference angle between the ego car and the center line of the lane, the smaller the reward value. That is, the vehicle will have a higher reward when driving along the reference center line value. For the positions and attitudes of the ego vehicle and the preceding/target vehicle, similar to the formula, the reward function is as follows, and the parameters in the formula are similar to those too:

(11) $$r_{t}^{ego\_obj\_1}=\{\begin{array}{c} b,S_{t}^{ego\_obj\_1}\in \left[d_{threshold}^{-},d_{threshold}^{+}\right]\\ -k_{1}\cdot S_{t}^{ego\_obj\_1},S_{t}^{ego\_obj\_1}>d_{threshold}^{+}\\ -k_{2}/S_{t}^{ego\_obj\_1},S_{t}^{ego\_obj\_1}<d_{threshold}^{+} \end{array}$$

In the above formula, the predetermined tracking distance range of the ego vehicle is $\left[d_{threshold}^{-},d_{threshold}^{+}\right]$. Similar to the actual vehicle driving process, the following distance will be enlarged along with the increase in the ego vehicle speed. After the introduction of the Frenet coordinate system, with the aim of maintaining a relatively stable attitude of both ego and target vehicles, the reward function also needs to be related to the projection ratio of the two velocities in the s direction.

(12) $$∥{\vec{v}}_{ego\_s}∥=clip(∥{\vec{v}}_{ego\_s}∥,v_{min},v_{max})\;∥{\vec{v}}_{obj\_s}∥=clip\left(∥{\vec{v}}_{obj\_s}∥,v_{min},v_{max}\right)\;r_{t}^{ego\_obj\_2}=k_{3}-clip\left(max\left(\frac{∥{\vec{v}}_{ego\_s}∥}{∥{\vec{v}}_{obj\_s}∥},\frac{∥{\vec{v}}_{obj\_s}∥}{∥{\vec{v}}_{ego\_s}∥}\right),value_{min},value_{max}\right)\;r_{t}^{ego\_obj}=r_{t}^{ego\_obj\_1}+r_{t}^{ego\_obj\_2}$$

In the formula, the projection of the speed of the ego vehicle and the target vehicle in the *s* direction are calculated, respectively, the maximum and minimum values are specified for the interception, and the relevant reward function design uses the ratio of the speeds of the ego vehicle and target vehicle. It can be seen from the above formula that when the discrepancy between the two is significant, the reward function is more minor, and only when the projection values of the ego vehicle and the target vehicle in the *s* direction are close or equal, the reward function is close to, or even reaches, the maximum value. In addition, as shown in the above state function, when the ego vehicle triggers the round termination condition in the round and does not reach the maximum number of training steps in the round, that is, when it exits the training under abnormal conditions, a large negative value needs to be added.

(13) $$r_{t}^{terminate}=\{\begin{array}{c} c,\mathrm{trigger}\;\mathrm{termination}\;\mathrm{condition}\\ 0,\mathrm{other} \end{array}$$

In addition to the above three reward functions, the discussion is also carried out according to the vehicle’s driving characteristics on straights and curves. On the straight road, the vehicle is supposed to eliminate the lateral offset, while on the curved road, especially when at a corner, the vehicle is expected to perform a large steering behavior in order to achieve cornering. Thence, the steer reward function can be expressed as follows:

(14) $$r_{t}^{steer}=\{\begin{array}{c} k_{4}steer_{ego},\mathrm{In}\;\mathrm{the}\;\mathrm{junction}\;\mathrm{area}\\ 0,\mathrm{other} \end{array}$$

So far, the reward function based on map constraints can be expressed as follows

(15) $$r_{t}=r_{t}^{ego\_wps}+r_{t}^{ego\_obj}+r_{t}^{terminate}+r_{t}^{steer}$$

#### 2.2.5. Simulation Results and Analysis

In the simulation process, a curve scene is intercepted for analysis. The trajectory and lateral distance are illustrated in the following figure.

**Figure 6:**
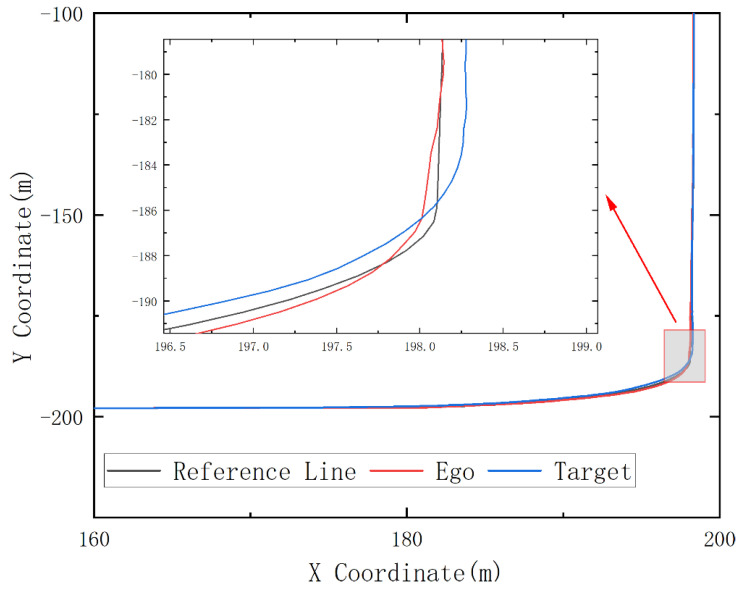
Turning process trajectory comparison chart.

PID is the abbreviation of “proportional–integral–derivative”, and PID is the most commonly used control method. Figure 6 shows the simulation results of the target vehicle controlled by PID and the ego vehicle controlled by reinforcement learning during the steering process. The target vehicle is driven by the traditional trajectory tracking control method, and the PID method is used in both the longitudinal and lateral directions of the target vehicle. The center line of the lane at the turning point is used as the trajectory and the preview point is extracted from it. In Figure 6, the black curve is the trajectory of the lane’s center line on the curve, while the red one represents the trajectory of the vehicle controlled by the reinforcement learning model at the turning point. The blue one represents the driving curve of the target vehicle using the PID method. According to the comparison curve of the simulation results, it is found that the reinforcement learning method achieved a more stable steering effect at the curving area than the target vehicle even though the former does not input the preview points like the PID method does.

**Figure 7:**
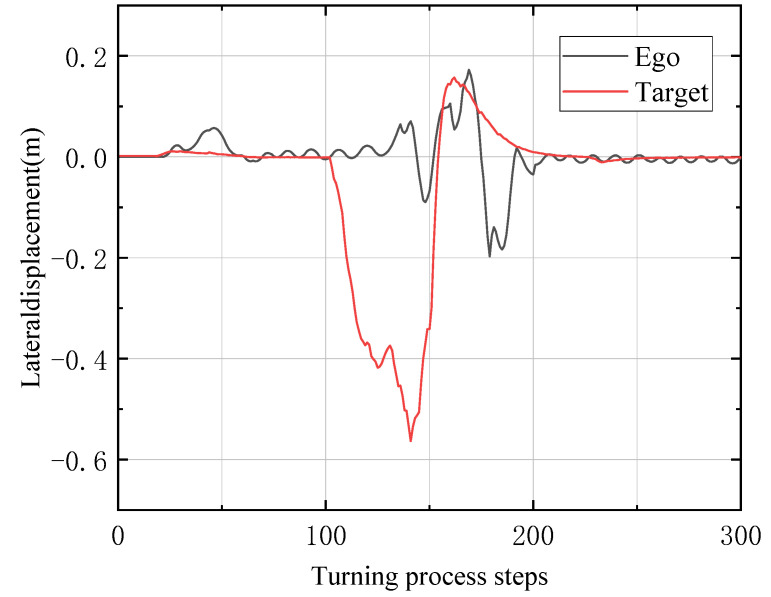
Comparison between the lateral displacement of the ego and target in the turning process.

In Figure 7, the black curve represents the ego vehicle controlled by reinforcement learning, and the red one refers to the target vehicle controlled by the traditional PID method. The x-coordinate is the number of time periods experienced in the steering process, and the y-coordinate is the lateral distance between the driving trajectories of the two vehicles and the lane center reference line. It can be seen from the curve results that the ego vehicle car controlled by reinforcement learning can also reach a relatively stable state at the turning point during the following process, and can fit the reference line well.

**Figure 8:**
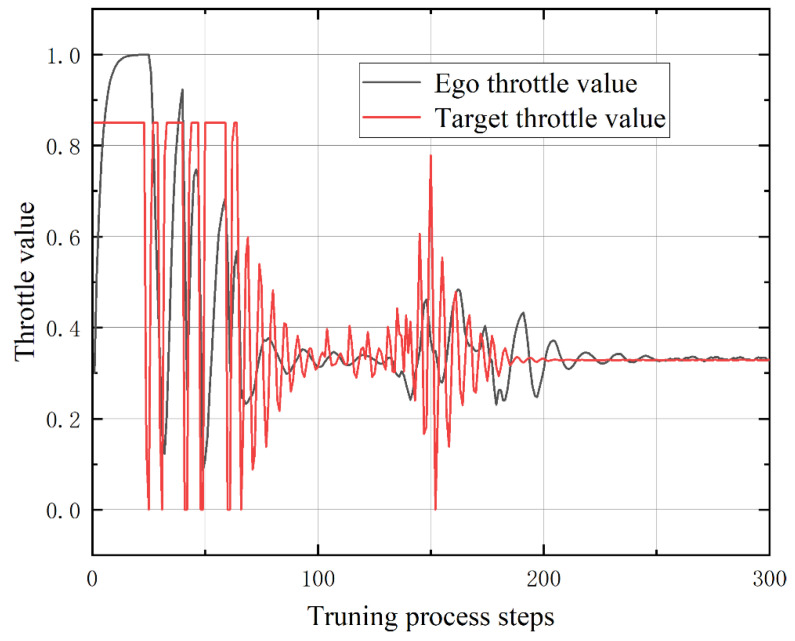
Comparison between throttle value of the ego and target in the turning process.

Figure 8 demonstrates the comparison of the throttle scalar input value during the turning process of the ego vehicle controlled by reinforcement learning and the target vehicle controlled by PID trajectory tracking. From the comparison of the figures, it can be seen that the throttle fluctuation range of the vehicle controlled by reinforcement learning is small, and the fluctuation range is relatively smooth. The trolley controlled by the PID method has a larger fluctuation range.

**Figure 9:**
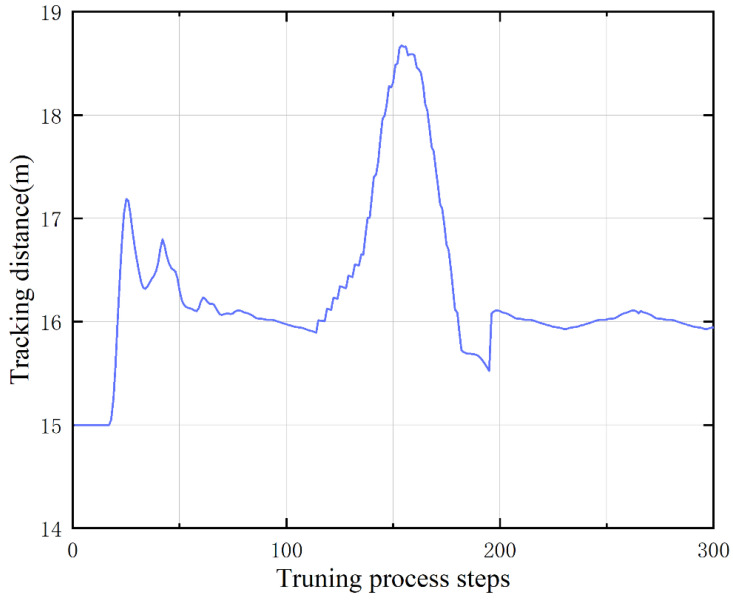
Tracking distance between the ego and target.

Figure 9 exhibits the projection of the distance between the ego vehicle and the target vehicle on the lane during the turning process. From the curve results, it is obvious that the following distance can be stabilized at 16 m on the straight road, and the distance at the turn has increased, and when it returns to the straight road again, the distance can also be restabilized at 16 m.

## 3. Obstacle Avoidance Using Deep Reinforcement Learning Approach

### 3.1. Improved Obstacle Representation

For the obstacle avoidance, owing to the fact that the number of external obstacles is not fixed, some researchers have adopted the rasterization processing method to rasterize the observation range. A rectangle grid or a sectorial grid is used to encode obstacles to determine the location and size of the obstacles. Moreover, the obstacle information is dimensionally reduced to address the issue that the input dimension is not fixed. For rasterization methods, a question that needs to be considered is the accuracy of the grid. The smaller the grid size, the higher the accuracy of its representation, and the larger the input dimension of the reinforcement learning neural network, which is more inconducive to episode training. However, if the grid size is enlarged, the accuracy will be worsened and the passable area will be shrunk, making it more difficult for the vehicle to get through the passable narrow area in complex obstacle avoidance scenarios. In a way, the grid map method will hinder the performance of the vehicle when passing through the narrow areas.

The perceptual information that the ego vehicle receives at a certain moment is unknown globally, and the perceptual results constantly change from time to time. In the case of uncertain external obstacles, taking the external obstacle input as the research object, it is difficult for us to utilize the fixed-dimensional data input to represent the obstacle information. Inspired by this limitation, it is thought that the shape of the ego vehicle is determined, and the ego vehicle’s position, attitude, and speed information can also be accurately obtained through the simulation platform or sensors. Suppose that a polygon is fixed on the vehicle body coordinate system. The polygon follows the movement of the vehicle, and the shape can change according to the speed of the vehicle. When the speed of the vehicle is slow, the polygon range is small. The extent of the polygon also expands in accordance with the increase in the vehicle’s speed. When the ego vehicle is driving in an obstacle avoidance scene, the state function of the obstacle input part only considers the part that enters the polygonal area. For the reinforcement learning neural network, this method can screen and sort out the complex obstacle scenes with unfixed numbers and the location distribution, but only a part of external obstacles within a certain range will be quantitatively calculated.

**Figure 10:**
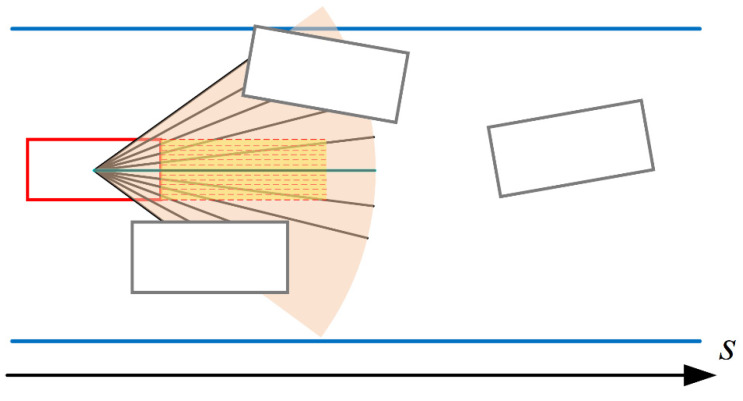
Schematic diagram of self-vehicle obstacle avoidance state input.

The improved state input of the self-vehicle obstacle avoidance process is shown in Figure 10. The input is divided into two areas, the sectorial area with the center of the ego vehicle as the dot and the extended rectangular area of the ego vehicle in the forward direction. The angle of the sector is determined by the maximum slip angle of the front wheels on both sides of the vehicle, while the width of the extended rectangular area depends on the bounding box length of the vehicle. Both areas take the center line of the vehicle’s forward direction as the axis. Among them, the sectorial area is divided by a group of equally angularly spaced line segments with the length of the sector radius. After encountering obstacles and lane boundary lines within the fan-shaped range, the corresponding inner-sector line segments will intersect with them. The data of line segments intersecting with the obstacles and lane boundary lines are stored as a list to represent the obstacle status within the sector. Similarly, the rectangular extension area of the yellow part is also divided by a cluster of equally spaced line segments. When obstacles and lane boundary lines are encountered and intersected with each other, the intersecting data of different interval line segments and obstacles are stored in a list to represent the status of obstacles in a rectangular area. The specific algorithm is shown in Algorithm 2:
**Algorithm** **2.** Obstacle input representation algorithmInitialize the sectorial and rectangular areas according to the maximum slip angle of the front wheel, the size of the ego vehicle frame, and the speed of the ego vehicle.Obtain the number $N_{sector}\;$ and $N_{rec}\;$ of the sectors and rectangles, and determine the angular interval of the sector β and the distance interval of the rectangle  ω.Combine multiple obstacle vehicles and lane boundaries into one obstacle set.Initialize the sector area representation $S_{sector}=\left[\left[\beta_{1},r\right],\cdots ,\left[\beta_{2},r\right]\right]$, and the rectangle area representation within the extended area $S_{rectangle}=\left[\left[\omega_{1},l\right],\cdots ,\left[\omega_{j},l\right]\right]$, in which r represents the radius of the sector and l represents the length of the rectangle.# Calculate sector representation.**For** $N_{sector}$ in cycles **do**: **if** The current line segment intersects the set of obstacles:  Confirm the intersection of the current line segment and the obstacle, and calculate the distance from the intersection to the origin, and update the corresponding $S_{sector}$ **else:**  The current $S_{sector}$ corresponding section is not updated**end for****for** $N_{rectangle}$ in cycles do: **if** The current line segment intersects the set of obstacles:  Confirm the intersection of the current line segment and the obstacle, and calculate the distance from the intersection to the corresponding horizontal reference line of the vehicle, and update the corresponding $S_{rectangle}$. **else**:  The current $S_{rectangle}$ corresponding section is not updated.**end for** Normalize the updated $S_{sector}$ and $S_{rectangle}$.

In the actual training process, the number of divisions of sectors and rectangles is set in advance by the parameter file, and the intervals are the same size, so the characterization of sectors and rectangles can be simplified as follows:

(16) $$\begin{array}{c} S_{sector}=\left[r_{1},r_{2},\cdots ,r_{N_{sector}}\right]\\ S_{rectangle}=\left[d_{1},d_{2},\cdots ,d_{Nrectangle}\right] \end{array}$$

As mentioned above, the lengths of the sectorial area and the rectangular area in the process are proportional to the speed of the ego vehicle:

(17) $$\begin{array}{c} r=k_{3}\cdot v_{ego}\\ l=k_{4}\cdot v_{ego} \end{array}$$

To represent the obstacle state at different ego vehicle speeds and calculate the reward function in the relevant state more accurately, the representation list in the region needs to be normalized, and the elements in $S_{sector}$ and $S_{sector}\;$ can be expressed as follows:

(18) $$\begin{array}{c} r_{i}=r_{i}/r\\ d_{i}=d_{i}/l \end{array}$$

Take Figure 10 as an example; the number of segmented rectangular areas is 10. After the algorithm and various optimizations, the representations in the sectorial area and the rectangular area are shown as

(19) $$\begin{array}{c} S_{sector}=\left[0.35,\;0.39,\;0.52,\;1,\;1,\;1,\;1,\;1,\;0.52,\;0.39,\;0.35\right]\\ S_{rectangle}=\left[1,\;1,\;1,\;1,\;1,\;1,\;1,\;1,\;1,\;1,\;1\right] \end{array}$$

On the left side and right side of the sectorial area, there is an intersection with the obstacle, and the simplified distance from the intersection to the center of the vehicle is as above. If the area near the center of the sectorial symmetry axis has no intersection with the obstacle, the corresponding state list element is 1. In the rectangular area, there is no intersection between the rectangular area and the obstacle in the current round, so the elements of the state list are all 1. From the current scene graph and this representation, it can be directly inferred in the current round that the ego vehicle can go straight forward from the current attitude.

### 3.2. Corresponding Reward Function Design

The sectorial areas and rectangular areas mentioned above are all forward detection areas during the driving process of the ego vehicle. The obstacles or lane boundaries in the detection area might affect the driving state of the ego vehicle in the following process. According to the schematic diagram of the obstacle avoidance scene and the corresponding state representation of obstacles and lane boundaries, it is found that the larger the element value, the farther the direction is from the obstacle or the lane boundary. When the element value is 1, it means that within the current detection range, there are no obstacles or lane boundaries in this direction. For the obstacles and lane boundaries within the detection range, the closer they are to the ego vehicle, the greater the possibility that the ego vehicle will collide with the obstacles or exceed the lane boundary; the closer the obstacles or lane boundary is to the forward direction of the ego vehicle, the higher the possibility that the ego vehicle will collide with an obstacle or exceed the lane boundary.

In order to describe the reward function of obstacles at different positions at each moment relative to the ego vehicle, the state bias vector and weight vector can be introduced. $B_{sector}$ represents the sectorial state bias vector and $B_{rectangle}$ represents the rectangle state bias vector. The role of bias vectors is to make the state vectors symmetrically distributed. During the calculation process of reward function in this part, the two-state bias vectors and the obstacle and lane boundary state functions are superimposed to generate new state vectors. The inner product of the new state vectors and the weight vectors obtained by this part of the operation are used as the benchmarks for the reward function of the obstacle and lane boundary parts, as described in the following formula,

(20) $$\begin{array}{c} B_{sector}=\left[b_{s\_1}\;b_{s\_2}\;\cdots \;b_{s\_N_{1}}\right]\\ W_{sector}=\left[w_{s\_1}\;w_{s\_2}\;\cdots \;w_{s\_N_{2}}\right] \end{array}$$

(21) $$r_{sector}=\left(S_{sector}-B_{sector}\right)\cdot W_{sector}^{T}=\left[x_{s_{1}}\;x_{s_{2}}\;\cdots \;x_{s_{N_{1}}}\right]\cdot \left[\begin{array}{c} w_{s_{1}}\\ \begin{array}{c} w_{s_{2}}\\ \cdots \end{array}\\ w_{s_{3}} \end{array}\right]=\overset{N_{1}}{\underset{i=1}{\sum }}x_{s\_i}\cdot w_{s\_i}$$

Among them, $W_{sector}$ is the weight vector of the sectorial area mentioned above. With the weight values, the closer to the center, the greater the weight, which means that the obstacle or boundary distance corresponding to the position has a greater impact on the driving of the ego vehicle. To obtain a large reward value, the ego vehicle tends to approach the driving state in which there are no obstacles and boundaries in the forward direction as much as possible. Similarly, the reward function in the rectangular area is as follows: $W_{rectangle}$ represents the weight vector of the rectangular area mentioned above. Different from $W_{sector}$, $W_{rectangle}$ represents the weight vector of the ego vehicle’s forward direction. As long as an external obstacle appears anywhere within the area, the vehicle will collide with the obstacle or exceed the lane boundary if it continues to go straight forward. Therefore, the weight of this area does now show much difference. There is little difference between the weights in this area.

(22) $$r_{rectangle}=\left(S_{sector}-B_{sector}\right)\cdot W_{sector}^{T}=\left[x_{r_{1}}\;x_{r_{2}}\;\cdots \;x_{r_{N_{1}}}\right]\cdot \left[\begin{array}{c} w_{r_{1}}\\ \begin{array}{c} w_{r_{2}}\\ \cdots \end{array}\\ w_{r_{3}} \end{array}\right]=\overset{N_{1}}{\underset{i=1}{\sum }}x_{r\_i}\cdot w_{r\_i}$$

In this obstacle representation method, the detection range is designed for different numbers of obstacles, and the input state of obstacles is unified, which is convenient to generate more complex data in the training process. Additionally, this method is used to unify the obstacle vehicles that need to be considered at each moment and the lane boundary lines on both sides into an obstacle set, which simplifies the separate discussion of obstacles and lane boundary modules in the obstacle avoidance process. This part of the reward replaces the reward section $r_{ego\_boundary}$ and $r_{ego\_obs}$, and the rest is the same as the previous section, which can be described as follows,

(23) $$\begin{array}{c} r_{ego\_obs}=r_{sector}+r_{rectangle}\\ r_{t}=r_{ego\_obs}+r_{ego\_other} \end{array}$$

### 3.3. Simulation Results and Analysis

**Figure 11:**
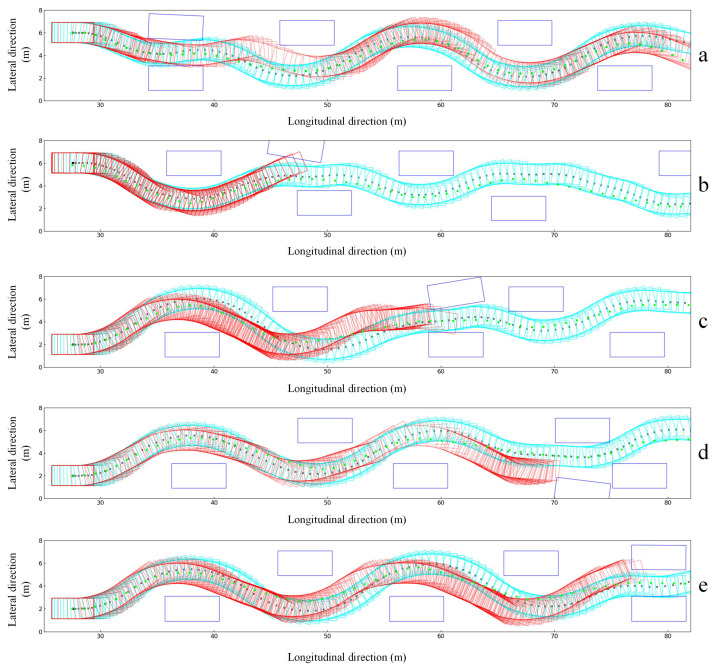
Trajectory comparison of three obstacle avoidance methods (**a**–**e**).

In the training process of the improved obstacle avoidance method, we enhance the environment complexity in the training process of the reinforcement learning neural network, which is embodied as follows: We have increased the randomness of the obstacle position and the randomness of the obstacle vehicle direction. In the obstacle environment of each episode, the scene of two vehicles side by side often occurs, resulting in a narrow traversable area at this location, and higher requirements for the attitudes of passing vehicles near this location. As shown in Figure 11, in these scenarios, under the model of the obstacle input state and reward function designed with the traditional method, the success rate of the ego vehicle passing through the area is low.

In order to compare with traditional classical methods, we also add the trajectory of the lattice planner into the same scenario. The lattice planner is a graph-based approach to the path planning issue that reduces the search space into a uniform discretization of vertices corresponding to positions and headings. It generates multiple obstacle avoidance trajectories, and then selects the most suitable one according to the cost ranking. The algorithm has good performance in both real vehicle and simulated obstacle avoidance scenes.

The trajectory comparison of the three obstacle avoidance methods is presented in Figure 11. The dark blue rectangle is obstacle vehicles on the road, and they are distributed in a random state within a specific range. The cyan one refers to the driving trajectory of the ego vehicle frame using the multi-obstacle input method, and the black points are the center locations of the ego vehicle during the whole episode. The green points represent the final trajectory points generated by the lattice planner. The red rectangle is the bounding trajectories of the ego vehicle using the reinforcement learning method with the traditional obstacle representation method. Specifically, in the first obstacle scene in the series, the side-by-side vehicles appear next to the first vehicle. Although the three obstacle avoidance methods all have passed the obstacle field in the simulation verification, the driving trajectories in the narrow area are slightly different. Similar to the traditional method lattice planner, the trajectory using the obstacle avoidance method with the obstacle state representation in Section 3 is smoother when passing through narrow areas, and the obstacle avoidance effect is more stable when compared with the ordinary obstacle avoidance method. It can be seen from the obstacle scenes in the series diagram that the ego vehicle, based on the common obstacle avoidance RL method, collided with obstacle vehicles. However, the obstacle avoidance method in Section 3 can still pass in a relatively stable state even though the traversable area is narrow.

Table 1 displays the statistics of the obstacle avoidance performance parameters of three methods. It is found that both our work and the lattice planner have better performance, but the lattice planner consumes a longer time per step and is more unstable. Although the common RL algorithm takes the shortest time and is relatively stable, it has limited ability of passing through complex obstacles. Our improved algorithm has the best and most stable passing performance, taking a relatively short time with the highest success rate.

From the results and obstacle avoidance trajectories of the above three obstacle avoidance methods, it can be seen that the improved multi-obstacle environment state and reward function design method can uniformly process the external obstacles with random numbers and attitudes so that the ego vehicle can better pass obstacle areas in complex obstacle scenarios.

## 4. Conclusions

In this paper, we first adopted the reinforcement learning method TD3 as the primary network, which was convenient for us to further adjust the relevant reward function in time according to the simulation training performance. The task scenarios of autonomous driving following and avoiding obstacles using the map constraints were investigated. For the following scenarios with turning roads, the state function and the corresponding reward function were designed in combination with the waypoint information in the map in the Frenet coordinate system. After 10,000 episodes of simulation training, the ego vehicle’s following performances on straights and corners reached a stable state.

Aiming at the multi-obstacle avoidance scene under the map constraints, we then proposed a representation method that could represent obstacles of different numbers and shapes. Combined with the obstacle representation method and the reinforcement learning model in the vehicle following scenario, the relevant reward function of the obstacle avoidance scene was designed. After a period of training, some obstacle scenes with narrow passable areas were constructed on the map. To verify the effect of two reinforcement learning methods, we compared them with the classic lattice planner. The results indicate that the obstacle representation method proposed by this paper has a better performance. In future work, we consider integrating the two single-task models into a multi-task reinforcement learning model.

In addition, multi-agent collaboration is also another research endpoint. We plan to focus on the reinforcement learning network model based on multi-agent collaboration using the map constraints. The cooperation among multi-agents is more in line with the future direction of intelligent networked vehicles.

## Figures and Tables

**Table 1:** Statistics of obstacle avoidance performance parameters of three methods under random difficulty conditions.

Algorithm	Our Work	Common RL	Lattice Planner
Average success rate (%)	95	70	92
Average calculation time (ms) Average time standard variance	3.42 0.37	2.96 0.31	35.41 10.48

## Data Availability

The data are not publicly available due to the nature of our laboratory for defense.
